# The Oral β-Lactamase SYN-004 (Ribaxamase) Degrades Ceftriaxone Excreted into the Intestine in Phase 2a Clinical Studies

**DOI:** 10.1128/AAC.02197-16

**Published:** 2017-02-23

**Authors:** John F. Kokai-Kun, Tracey Roberts, Olivia Coughlin, Eric Sicard, Marianne Rufiange, Richard Fedorak, Christian Carter, Marijke H. Adams, James Longstreth, Heidi Whalen, Joseph Sliman

**Affiliations:** a Synthetic Biologics, Inc., Rockville, Maryland, USA; b Algorithme Pharma, Laval, Quebec, Canada; c University of Alberta/Alberta Health Service, Edmonton, Alberta, Canada; d MH Adams & Associates, Inc., Davie, Florida, USA; e Longstreth and Associates, Inc., Mundelein, Illinois, USA

**Keywords:** beta-lactamases, ceftriaxone, clinical trials, dysbiosis, gut microbiome, oral administration, protection

## Abstract

SYN-004 (ribaxamase) is a β-lactamase designed to be orally administered concurrently with intravenous β-lactam antibiotics, including most penicillins and cephalosporins. Ribaxamase's anticipated mechanism of action is to degrade excess β-lactam antibiotic that is excreted into the small intestine. This enzymatic inactivation of excreted antibiotic is expected to protect the gut microbiome from disruption and thus prevent undesirable side effects, including secondary infections such as Clostridium difficile infections, as well as other antibiotic-associated diarrheas. In phase 1 clinical studies, ribaxamase was well tolerated compared to a placebo group and displayed negligible systemic absorption. The two phase 2a clinical studies described here were performed to confirm the mechanism of action of ribaxamase, degradation of β-lactam antibiotics in the human intestine, and were therefore conducted in subjects with functioning ileostomies to allow serial sampling of their intestinal chyme. Ribaxamase fully degraded ceftriaxone to below the level of quantitation in the intestines of all subjects in both studies. Coadministration of oral ribaxamase with intravenous ceftriaxone was also well tolerated, and the plasma pharmacokinetics of ceftriaxone were unchanged by ribaxamase administration. Since ribaxamase is formulated as a pH-dependent, delayed-release formulation, the activity of ribaxamase in the presence of the proton pump inhibitor esomeprazole was examined in the second study; coadministration of these drugs did not adversely affect ribaxamase's ability to degrade ceftriaxone excreted into the intestine. These studies have confirmed the *in vivo* mechanism of action of ribaxamase, degradation of β-lactam antibiotics in the human intestine (registered at ClinicalTrials.gov under NCT02419001 and NCT02473640).

## INTRODUCTION

A balanced gut microbiome is important for human health (1, 2), and disruption of this balance has been associated with various maladies from opportunistic infections like Clostridium difficile (3–7) to metabolic syndromes and neurologic disorders (1, 2, 8–11). Although a number of factors can disrupt the balance of the gut microbiome, leading to a state of dysbiosis (12, 13), one of the most significant factors is the use of antibiotics (3, 6, 14–16). Antibiotics can reduce or eliminate commensal bacterial populations in the gut which then, due to the loss of colonization resistance, allows outgrowth of potentially pathogenic organisms (3, 15). This is particularly true for C. difficile infections, where a primary risk factor is repeated exposure of the gut microbiome to antibiotics (3–7, 17, 18).

Antibiotic use also leads to the potential selection of antibiotic-resistant organisms (19–21). When the diverse population of the gut microbiome is exposed to antibiotics, emergence of resistance can occur through several mechanisms (22). The antibiotics may directly select for antibiotic-resistant variants of enteric pathogens that inhabit the human colon in low numbers (23) or eliminate the colonization resistance that keeps these organisms at bay (24). Similarly, antibiotic-mediated selection of resistant commensal organisms may result in transfer of resistance genes to pathogenic organisms in the gut (25).

The β-lactam antibiotics (cephalosporins, penicillins, and carbapenems) are highly effective antibiotics for treating bacterial infections, but they can also be highly disruptive to the gut microbiome (4, 5, 7) and can lead to the selection of antibiotic-resistant pathogens (23, 25). This occurs even when these antibiotics are delivered intravenously (i.v.), since a substantial portion of the dose can be excreted in the bile and reach the intestine as a fully functional antibiotic (26–28). This is particularly true for ceftriaxone, a highly effective third-generation cephalosporin, for which more than half of the i.v. dose can be excreted through the bile into the intestine and result in disruption of the gut microbiome (27, 28). Although antibiotics remain essential for the treatment of bacterial infections, strategies are clearly needed to protect the healthy gut microbiome and prevent the emergence of antibiotic resistance.

SYN-004 (ribaxamase) is a β-lactamase which is designed to be orally administered concurrently with certain i.v. β-lactam antibiotics (such as ceftriaxone). Ribaxamase is a modified version of a previously developed class A β-lactamase (P1A, a naturally occurring penicillinase isolated from Bacillus licheniformis, the PenP protein [29–33]), which has been engineered through a single amino acid substitution (Asp 247 [Ambler 276] to Asn) to extend its β-lactam degradation spectrum to include later-generation cephalosporins (34). Ribaxamase is manufactured as a pH-dependent formulation for release in the proximal small intestine (34) and has been shown not to affect the plasma pharmacokinetics of i.v. ceftriaxone in dogs (35). Ribaxamase was also well tolerated in two phase 1, placebo-controlled clinical studies in 64 normal, healthy volunteers in which single doses (up to 750 mg) and multiple doses (up to 300 mg every 6 h for 7 days) were administered (36). In these phase 1 studies, systemic absorption of ribaxamase was sporadic and negligible, with no anti-enzyme antibodies detected (36).

The presently described open-label, randomized phase 2a studies enrolled subjects with functioning ileostomies, who were otherwise healthy, to allow facile serial sampling of their intestinal chyme. In these studies, subjects were administered i.v. ceftriaxone alone or with oral ribaxamase and in the absence or presence of the proton pump inhibitor (PPI) esomeprazole. Serial samples of chyme and plasma were analyzed for concentrations of ceftriaxone and ribaxamase to confirm the *in vivo* mechanism of action of the enzyme and to examine its capacity to degrade ceftriaxone excreted into the intestine.

(Parts of this work were presented at ASM Microbe, 2016, and IDWeek, 2016.)

## RESULTS

### Subjects.

Study 1 enrolled 11 subjects, while study 2 enrolled 15 subjects; 3 subjects participated in both studies. Of the 11 subjects enrolled in study 1, 6 subjects were randomly assigned to receive 75 mg of ribaxamase, and 5 subjects were assigned to receive 150 mg of ribaxamase. Ten of 11 subjects completed the study. One of 6 subjects from the assigned 75 mg group prematurely discontinued study participation during period 1 due to an adverse event (AE; a ceftriaxone infusion-related reaction, not related to ribaxamase administration). In study 2, 14 of the 15 subjects completed the study, while one subject prematurely discontinued study participation due to an AE (stoma site hemorrhage related to ceftriaxone and ribaxamase administration). The demographics for the subjects enrolled in the two studies are presented in Table S1 in the supplemental material. All 23 unique subjects in both studies had at least one prior medical history finding and, in addition to the expected findings of surgical procedures related to the placement of an ileostomy, 11 subjects had been diagnosed with Crohn's disease and 7 subjects with ulcerative colitis.

### Safety assessments.

There were no deaths or serious AEs reported during either study and, other than the one subject from each study that discontinued, as described above, no other subjects discontinued either study. No subject in either study had clinically significant after baseline changes in laboratory results or a new abnormal physical exam result. Mean and median electrocardiogram parameters also did not differ between periods in either study. Mean and median vital signs were similar in both periods in both studies and not significantly different from predose values in either study, except for one subject in study 2 who had a potentially clinically significant change in pulse rate that was unrelated to study drug administration. This subject completed the study.

In study 1, a total of 12 treatment-emergent adverse events (TEAEs; occurring in 7 of 11 subjects) were reported that were considered related to ribaxamase or ceftriaxone (see Table S2 in the supplemental material). A total of 6 of 11 subjects had TEAEs considered related to ceftriaxone alone during period 1 when no ribaxamase was administered, and a total of 3 of 11 subjects (2 of 6 in the 75-mg group and 1 of 5 in the 150-mg group) had TEAEs considered related to ribaxamase and ceftriaxone during period 2. The only TEAE that occurred in more than one subject was headache.

In study 2, a total of 8 TEAEs (occurring in 5 of 15 subjects) were considered related to any study drug (see Table S2 in the supplemental material). In period 1, 4 of 15 subjects had 7 TEAEs, and 6 of these were considered by the investigator to be related to both ribaxamase and ceftriaxone since both drugs were administered during the period. During the esomeprazole run-in period, one subject had a TEAE considered related to esomeprazole (headache), and no TEAEs were reported during ceftriaxone plus ribaxamase administration in the presence of esomeprazole in period 2.

### Pharmacokinetics. (i) Plasma concentrations.

**FIG 1:**
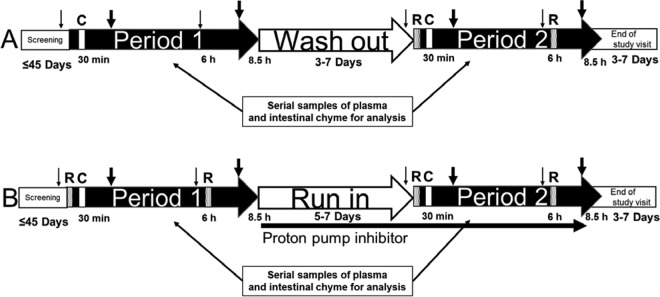
Study design for the two clinical studies conducted in subjects with ileostomies. (A) Study 1; (B) study 2. Patients were screened for the studies up to 45 days prior to period 1. An intravenous infusion (30-min infusion) of ceftriaxone (1 g) was administered at 30 min of each period as indicated (C), and oral ribaxamase (75 or 150 mg in study 1 and 150 mg in study 2) was administered at 0 min and 6 h as indicated (R). Small nonfatty meals were provided at 1 h prior to and 5 h after the infusion (small arrows) and full meals at 2 and 7.5 h postinfusion (thick arrows). Subjects were required to drink water or apple juice during periods 1 and 2 to promote intestinal chyme production. A washout period of 3 to 7 days separated period 1 and period 2 of study 1, and in study 2, a run-in period of 5 to 7 days occurred during which subjects self-administered 40 mg of oral esomeprazole daily in the morning. In study 2, subjects also received 40 mg of esomeprazole 1 h prior to the first dose of ribaxamase in period 2. In both studies, serial plasma and chyme samples were collected for analysis during periods 1 and 2, and an end-of-study visit occurred 3 to 7 days after period 2.

**FIG 2:**
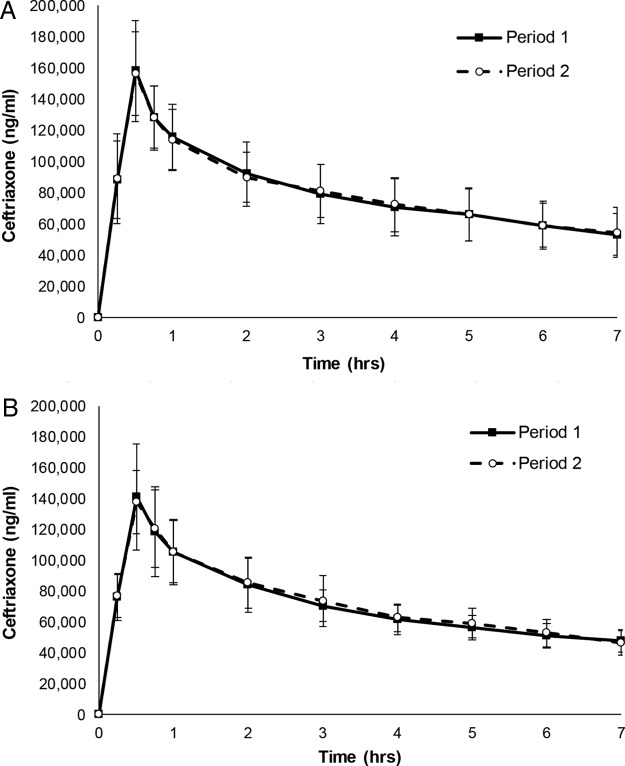
Comparison of ceftriaxone concentrations in plasma in periods 1 and 2 in the two studies. The graphs display the mean plasma ceftriaxone concentration (± the standard deviations [SD]) over time for 10 subjects from study 1 (A) and 14 subjects from study 2 (B). Study 1 period 1, i.v. ceftriaxone only; period 2, i.v. ceftriaxone + oral ribaxamase. Study 2 period 1, i.v. ceftriaxone + oral ribaxamase; period 2, i.v. ceftriaxone + oral ribaxamase + esomeprazole. The 0-h time points on the graphs represent the ceftriaxone i.v. infusion start.

Of the 11 subjects enrolled in study 1 and 15 subjects enrolled in study 2, 10 and 14 subjects, respectively, completed both periods in each study and were considered evaluable for pharmacokinetics (PK) (Fig. 1). In periods 1 and 2 of both studies, all plasma samples other than the predose samples contained measurable concentrations of ceftriaxone. In each ribaxamase dose group in study 1 (75 or 150 mg) and in study 2, the mean ceftriaxone *C*_max_ values were similar (i.e., within 1 standard deviation [SD]) in periods 1 and 2. The maximum concentration of ceftriaxone occurred at 0.5 h for all subjects, as expected, since this sample time corresponded with the end of the 30-min infusion. The pharmacokinetic parameters for ceftriaxone in plasma for both studies are presented in Table 1. Mean ceftriaxone concentration versus time profiles in plasma were superimposable after a 1-g i.v. infusion with or without concomitant oral ribaxamase administration in study 1 (Fig. 2A) and with concomitant oral ribaxamase administration in the absence or presence of PPI in study 2 (Fig. 2B). Similarities in the concentration profiles for periods 1 and 2 were also seen within all individual subjects in both studies (data not shown). Despite the short sampling interval, the *R*^2^ value was ≥0.899 for all regression lines for individual subjects in both studies. The concentration of ribaxamase in plasma was below the level of quantitation (0.8 ng/ml) for all samples collected during period 2 of study 1 for both doses (75 and 150 mg) of ribaxamase.

**TABLE 1:** Pharmacokinetic parameters of ceftriaxone in plasma in studies 1 and 2

Study	Ribaxamase dose (mg)	Period	Mean ± SD^*b*^	
			*C*_max_ (ng/ml)	AUC*_t_* (h·ng/ml)
1	0	1^*a*^	137,800 ± 10,710^c^	464,400 ± 26,884
	75	2	143,800 ± 13,103	478,800 ± 37,117
	0	1	178,600 ± 34,623	625,200 ± 105,257
	150	2	169,200 ± 32,782	643,800 ± 108,349
2	150	1	141,500 ± 34,482	497,700 ± 81,869
	150	2	138,000 ± 20,591	507,100 ± 84,861

a No ribaxamase was administered.

b *C*_max_ is the peak plasma concentration; AUC*_t_* is the area under the plasma concentration-time curve from time zero to the last quantifiable concentration.

### (ii) Chyme concentrations.

**FIG 3:**
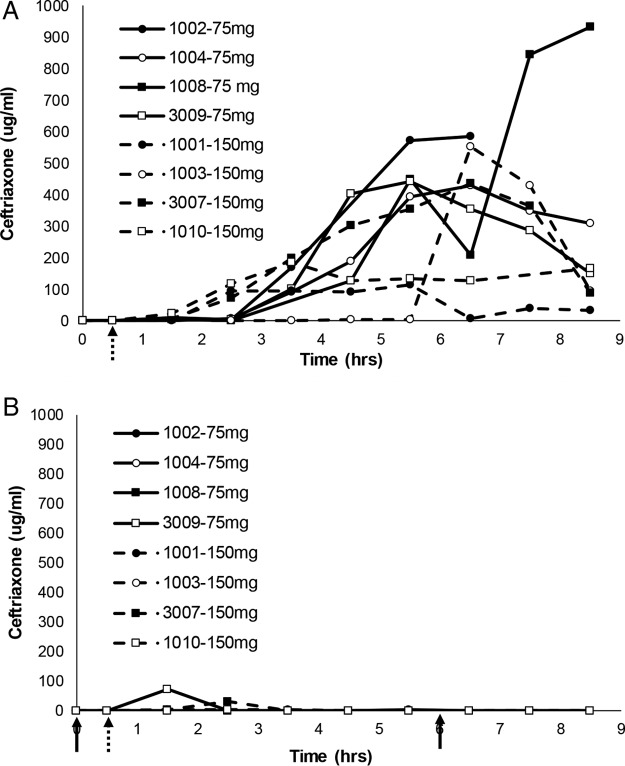
Comparison of ceftriaxone concentrations in intestinal chyme for eight subjects from study 1. Serial chyme samples (as available) were collected and analyzed for their ceftriaxone concentrations over time in periods 1 (A) and 2 (B) of study 1. The data show individual ceftriaxone concentration curves (assay lower limit of quantitation, 1 μg/ml) for eight subjects (subject numbers indicated on the figure) over time. The 30-min i.v. ceftriaxone (1 g) infusion began at the hatched arrow, while 75 or 150 mg (as randomized and indicated on the figures) of oral ribaxamase was administered at the solid arrows in period 2. Missing data points indicate that no chyme was available for collection at that time point. Two subjects from this study were excluded from this figure (see the text and Fig. S1 in the supplemental material).

In study 1, ceftriaxone concentrations in chyme were below the lower limit of quantitation (BLQ) until at least 1.5 h after starting the infusion. During period 1 (Fig. 3A), measurable concentrations of ceftriaxone then ranged from 1.01 to 1,345 μg/ml during the sample collection interval. For both ribaxamase dose groups (75 and 150 mg), the mean ceftriaxone concentration-time profiles in chyme were substantially lower in period 2 (Fig. 3B) than in period 1 from ∼4 h after the start of the ceftriaxone infusion (∼4.5 h after the first ribaxamase dose) through to the last samples collected for 8 of the 10 subjects, indicating that ceftriaxone in the intestine was effectively degraded by the oral ribaxamase administered in period 2. The mean ceftriaxone concentrations in chyme at the later time points were generally higher (when above the lower limit of quantitation [LLOQ]) for the 75-mg ribaxamase dose group (median at 5.5 h = 1.46 μg/ml) than for the 150-mg ribaxamase dose group (median at 5.5 h = 0.0 μg/ml), suggesting an additional effect with the higher dose.

**FIG 4:**
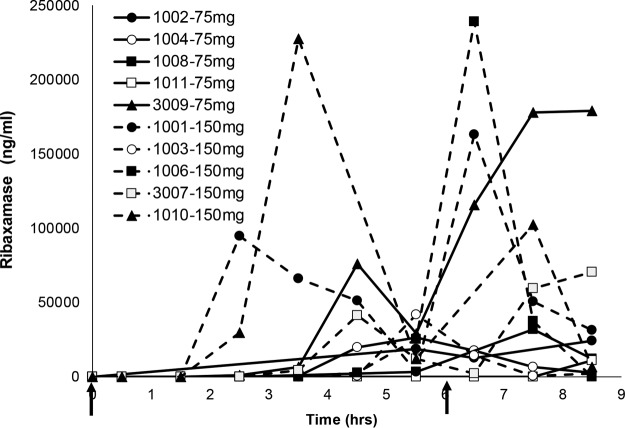
Ribaxamase concentrations in intestinal chyme for ten subjects from period 2 of study 1. Serial chyme samples (as available) were collected and analyzed for their ribaxamase concentrations over time in period 2 of study 1. The data show individual concentration curves (assay LLOQ, 10 ng/ml) for 10 subjects (subject numbers are indicated in the figure) over time. Two doses of oral ribaxamase were administered (75 or 150 mg, as randomized and indicated on the figure) at the solid arrows. Missing data points indicate that no chyme was available for collection at that time point.

In study 1, concentrations of ribaxamase in chyme were BLQ until at least 1.5 h after starting the ceftriaxone infusion (at least 2 h after the first dose of ribaxamase; Fig. 4) and all subjects had quantifiable concentrations of ribaxamase in their chyme by the 8.5-h time point (2.5 h after the second dose of ribaxamase). Measurable concentrations of ribaxamase were in the range of 29 to 239,000 ng/ml during the sample collection interval. The concentrations of ribaxamase in chyme were generally higher for the 150-mg dose group than for the 75-mg dose group (Fig. 4).

Even though the subjects were on a standardized diet and encouraged to drink fluids during the study, ribaxamase concentrations in chyme were variable (Fig. 4). Two of the subjects in study 1 seemed to have delayed appearance of ribaxamase in their chyme during period 2 compared to the other eight subjects (subjects 1006 [see Fig. S1A in the supplemental material] and 1011 [see Fig. S1B]), and this delay in the appearance of ribaxamase corresponded to higher concentrations of ceftriaxone in these subjects' chyme until 4 and 8 h, respectively, after the first dose of ribaxamase. When ribaxamase was detected in these subjects' chyme at 4 and 8 h, however, the concentration of ceftriaxone dropped to BLQ for both subjects (Fig. S1A and B).

**FIG 5:**
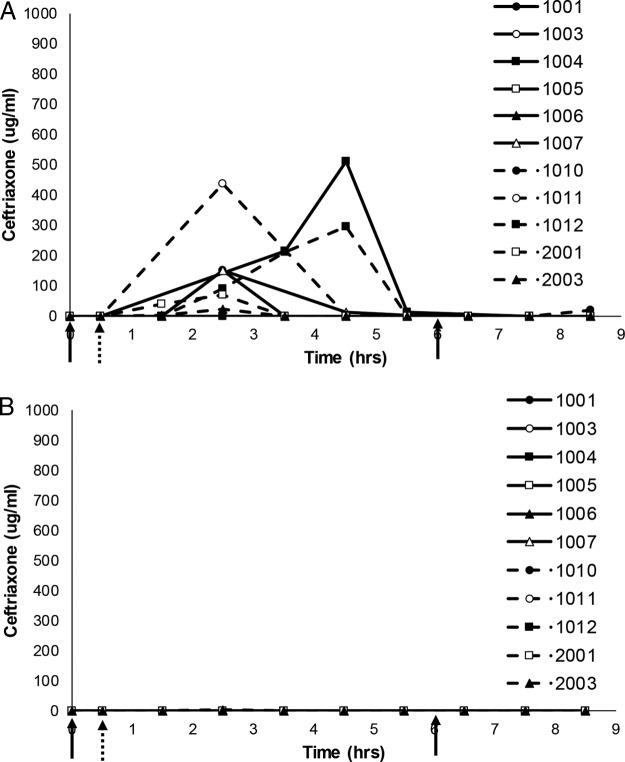
Comparison of ceftriaxone concentrations in intestinal chyme for eleven subjects from study 2. Serial chyme samples (as available) were collected and analyzed for their ceftriaxone concentrations over time in periods 1 (A) and 2 (B) of study 2. The data show individual ceftriaxone concentration curves (assay LLOQ, 1 μg/ml) for 11 subjects (subject numbers are indicated in the figure) over time. The 30-min ceftriaxone (1 g) infusion began at the hatched arrow, while 150 mg of oral ribaxamase was administered at the solid arrows. Period 2 was when esomeprazole was present. Missing data points indicate that no chyme was available for collection at that time point. Three subjects from this study were excluded from this figure (see the text and Fig. S2 in the supplemental material).

In study 2 during both periods, ceftriaxone concentrations in chyme, when detectable, were also BLQ until at least 1.5 h after starting the i.v. infusion (Fig. 5) and were BLQ in 79% (177/226) of the chyme samples collected during the study. Over both periods, only 21% (48/226) of the collected chyme samples had measurable ceftriaxone concentrations, and these were generally lower than those seen in period 1 of study 1 when ceftriaxone was administered without any ribaxamase (Fig. 3A). In period 2 of study 2 (Fig. 5B, with concomitant esomeprazole) compared to period 1 (Fig. 5A), fewer samples had measurable concentrations of ceftriaxone (11% versus 23%), and more samples had concentrations BLQ (71% versus 56%). Measurable ceftriaxone concentrations in chyme were lower in period 2 after multiple doses of esomeprazole (range, 1.04 to 561 μg/ml; median, 13.2 μg/ml) than in period 1 (range, 1.06 to 1,205 μg/ml; median, 35.3 μg/ml). The mean ceftriaxone concentration-time profiles were substantially lower in period 2 than in period 1 from ∼2.5 h after the start of the ceftriaxone infusion (∼3 h after the first ribaxamase dose) through to the last samples collected (Fig. 5), indicating the daily doses of esomeprazole administered before and during period 2 had no negative effect on ribaxamase activity.

Similar to what was seen in study 1, two subjects in study 2 (1002 and 1008) had delayed appearance of ribaxamase in their chyme during period 1, and this corresponded to higher concentrations of ceftriaxone prior to the appearance of ribaxamase (see Fig. S2A and C). In period 2, however, ribaxamase was detected 3 h earlier for subject 1002 and 1 h earlier for subject 1008 both of which corresponded to earlier degradation of ceftriaxone in this period for these two subjects (see Fig. S2B and D). For unknown reasons, one subject (subject 1009) had barely detectable levels of ribaxamase in periods 1 and 2 of the study, with concentrations only reaching peaks of 2.1 and 0.91 μg/ml, respectively, but even at these low concentrations of ribaxamase the concentration of ceftriaxone was BLQ around the time points in which any concentration of ribaxamase was detected (data not shown).

**FIG 6:**
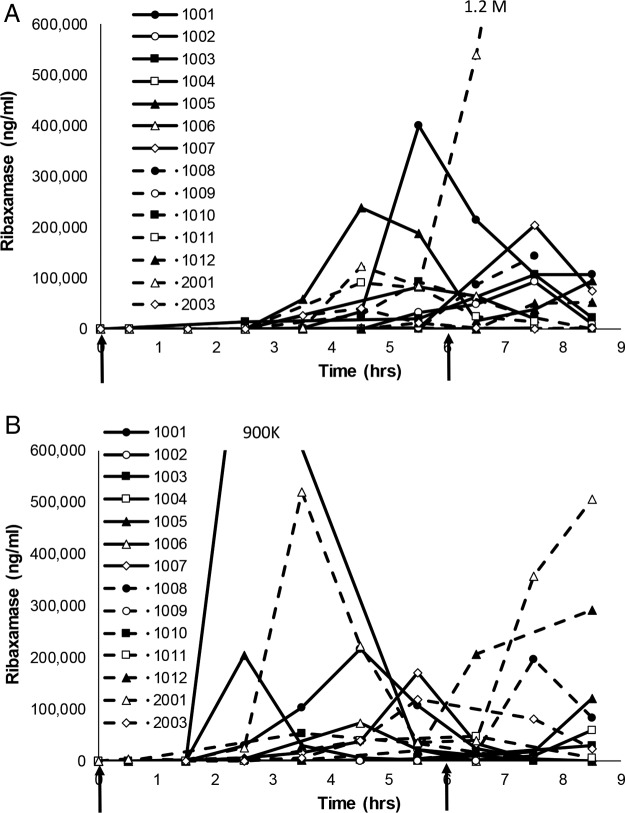
Ribaxamase concentrations in intestinal chyme for fourteen subjects from study 2. Serial chyme samples (as available) were collected and analyzed for their ribaxamase concentrations over time in hours in periods 1 (A) and 2 (B) of study 2. The data show individual concentration curves (assay LLOQ, 10 ng/ml) for 14 subjects (subject numbers are indicated in the figure) over time. Two doses of oral ribaxamase were administered (150 mg/dose) at the solid arrows. Missing data indicate that no chyme was available for collection at that time point. Period 2 is when esomeprazole was present. Both graphs were truncated for clarity, and the numbers indicate the peak concentrations for the two subjects with truncated profiles.

The ribaxamase concentrations in chyme were measurable in at least one subject in each period of study 2 by 0.5 h after starting the ceftriaxone infusion, which was 1 h after the first ribaxamase dose (Fig. 6). The mean concentration-time profiles for ribaxamase in both periods were similar (i.e., mean values within 1 SD of each other), but in period 2 ribaxamase was generally detected earlier in the chyme than for the same subjects in period 1 (Fig. 6).

Three of the subjects participated in both studies (which occurred several months apart), and a comparison of their ribaxamase concentrations in chyme for study 1, period 2, and study 2, period 1, are presented in Fig. S3 in the supplemental material. Two of the subjects (study 1/study 2: 1006/1002 and 3009/2001) had similar temporal appearances of ribaxamase in their chyme but with substantially different concentrations. Ribaxamase appearance was similarly delayed for Subject 1006/1002 in both studies compared to most of the other subjects in period 2 of study 1 and period 1 of study 2 (Fig. 4 and 6). Subject 1006/1002 received 150 mg of ribaxamase in both studies, while subject 3009/2001 received 75 mg of ribaxamase in study 1 and 150 mg in study 2. Subject 1001/1001 received 150 mg of ribaxamase in both studies but had detectable ribaxamase in their chyme about 2 h earlier in study 1 period 2 than in study 2 period 1 and also had different concentrations of ribaxamase detected in the two studies.

## DISCUSSION

Ribaxamase is a β-lactamase designed to be orally administered during treatment with certain i.v.-administered β-lactam antibiotic to protect the gut microbiome from disruption by intestinally excreted antibiotics. Ribaxamase possesses the properties necessary to be an effective protectant of the gut microbiome, which were demonstrated in preclinical studies (34), nonclinical studies (35), and phase 1 clinical studies, which also demonstrated that ribaxamase was well tolerated compared to the placebo and was not systemically absorbed in humans (36). The present phase 2a clinical studies were designed to confirm the mechanism of action of ribaxamase, degradation of β-lactam antibiotics, in the human intestine. To evaluate this mechanism of action, a unique subject population was recruited for these studies, subjects with functioning ileostomies. By using these subjects, serial samples of chyme could be obtained in a minimally invasive manner.

These studies demonstrated that the combination of oral ribaxamase and i.v. ceftriaxone was well tolerated (see Table S2 in the supplemental material) and confirmed that while ribaxamase effectively degraded ceftriaxone excreted into the human intestine (Fig. 3), it did not significantly affect the systemic PK of the i.v.-administered antibiotic (Fig. 2). Although ribaxamase degraded ceftriaxone in the human intestine when the enzyme was present, there was also variability observed in both excretion of ceftriaxone into the intestine (Fig. 3 and 5), as well as the appearance of ribaxamase in the intestine (Fig. 4 and 6). This occurred even though all subjects in the two studies were required to fast overnight prior to dosing and were served standard meals on a standard schedule (Fig. 1). This variability was seen even between studies for the three subjects who participated in both studies (see Fig. S3). Conversely, the plasma PK for ceftriaxone across both studies in all subjects were relatively consistent (Fig. 2). The variability of the concentration curves for ceftriaxone and ribaxamase in the chyme was likely a result of differences in stomach emptying, chyme production, and bile secretion from the gallbladder, and possibly also a result of the location of the ileostomy in the subjects, and is consistent with what was seen in preclinical studies with ribaxamase in dogs (34). Because of the variability seen in the concentration curves for ribaxamase in intestinal chyme, the 150-mg dose of ribaxamase, administered every 6 h during antibiotic dosing (and for a short time thereafter), has been selected for continued clinical development to provide a sufficient concentration of ribaxamase to degrade ceftriaxone even if ceftriaxone is excreted sporadically into the intestine.

PPIs are commonly administered to hospitalized patients and have the effect of raising the pH of the stomach and upper intestine (37) and modifying the intragastric release of other drugs from their dosage forms (38). Ribaxamase uses a pH-dependent formulation to protect the protein from the stomach acidity, and this formulation is designed to release active enzyme when the pH exceeds 5.5, which is predicted to be proximal to the ampulla of Vater, where the pancreatic duct and bile duct join together to drain into the duodenum. It is anticipated that this point of release for active enzyme will result in inactivation of antibiotic excreted via the bile after i.v. administration. Since the release of ribaxamase is pH dependent, the formulation was studied in the ileostomy population in the presence of a PPI. Study 2 (Fig. 5) clearly showed that esomeprazole administration has no negative effect on the ability of ribaxamase to degrade ceftriaxone and seems to have initiated the degradation of ceftriaxone in chyme earlier after administration. This is most likely due to a more consistent, somewhat higher pH level in the small intestine leading to an earlier release of active enzyme. Even in the presence of a PPI, however, there was some variability in the appearance of ribaxamase in the intestine (Fig. 6B). These observations, however, are not critical to the use of ribaxamase since, once a steady-state of enzyme is established with 6-h dosing, the active enzyme, which has been shown to be stable for at least 6 h in human chyme and 8 h in the dog intestine (34), should be continually present in the intestinal tract to degrade residual antibiotic excreted into the intestine during and after i.v. dosing.

Care was taken with the chyme samples to ensure that postsampling ceftriaxone degradation was inhibited in the samples immediately after collection. This was accomplished by flash freezing the chyme samples and then thawing the samples on ice and extracting the samples for ceftriaxone analysis with 8 M guanidine to inactivate any enzyme in the samples prior to analysis. Thus, the degradation of ceftriaxone measured during both studies should reflect what is occurring in the human intestine.

During the selection of a neutralizer of β-lactamase activity in isolated chyme, various β-lactamase inhibitors, such as sulbactam and tazobactam, which are effective for the inhibition of a class A serine β-lactamase such as ribaxamase (39), were investigated. Even at their maximum concentrations based on solubility, however, these β-lactamase inhibitors could not block the enzymatic activity of ribaxamase at the higher concentrations found in the chyme (data not shown). This finding supports that ribaxamase should also have utility when used with β-lactam/β-lactamase inhibitor combinations such as piperacillin-tazobactam and possibly newer combinations such as ceftolozane/tazobactam and is consistent with clinical data from the progenitor P1A β-lactamase which demonstrated that this enzyme was effective for neutralization of piperacillin-tazobactam in the human intestine (31).

In summary, data from these two clinical studies support the expected mechanism of action of ribaxamase, which is to degrade certain β-lactam antibiotics excreted into the intestine. In these studies, ribaxamase was also found to be well tolerated when administered with i.v. ceftriaxone and not affect the plasma PK of the antibiotic. These findings supported the advancement of ribaxamase into a recently concluded double-blind, placebo-controlled phase 2b clinical study designed to assess the ability of ribaxamase to prevent C. difficile-associated disease, antibiotic-associated diarrhea, colonization by opportunistic enteric pathogens, and the emergence of antibiotic resistance by protecting the gut microbiome from the deleterious effects of ceftriaxone excreted into the intestine.

## MATERIALS AND METHODS

### Study drugs.

Ribaxamase, an enzyme of ∼29 kDa, was expressed in a recombinant Escherichia coli system and then purified to near homogeneity by Fujifilm-Diosynth Biotechnologies UK (Billingham, United Kingdom). The purified ribaxamase bulk protein was formulated as an oral capsule containing enteric-coated pellets designed to release active enzyme within the intestine once the pellets reached a region of the intestine where the pH was above 5.5 (34). Each capsule contained 75 mg of active ribaxamase enzyme. Ceftriaxone sodium (1 g) was obtained from commercial sources and prepared for i.v. administration by the site pharmacists. Esomeprazole magnesium (Nexium delayed-release capsule formulation [AstraZeneca], 40 mg) was obtained from commercial sources.

### Study designs.

Two phase 2a randomized, multicenter, open-label clinical studies were conducted in a total of 26 subjects with functioning ileostomies who were otherwise healthy. This subject population allowed serial sampling of their intestinal chyme. Both studies were performed in accordance with the Declaration of Helsinki as revised in 2013 and under Local Ethics Committee approval.

The first study (study 1, NCT02419001, Fig. 1A) evaluated the ability of orally administered ribaxamase to degrade ceftriaxone in the small intestine after i.v. ceftriaxone administration. The degradation of ceftriaxone in the small intestine was monitored by quantifying intact antibiotic concentrations in the intestinal chyme. In this study, 1 g of ceftriaxone was administered i.v. without (in period 1) or with (in period 2) two oral doses (given 6 h apart) of 75 or 150 mg of ribaxamase. Concentrations of ceftriaxone were evaluated in collected chyme and plasma samples from both periods, while ribaxamase concentrations were evaluated in both matrices in period 2.

The second study (study 2, NCT02473640, Fig. 1B) evaluated the ability of ribaxamase to degrade ceftriaxone in the presence of a PPI, since ribaxamase is pH-dependent formulation. This study was similar to study 1, except that subjects received both ribaxamase (150 mg) and ceftriaxone (1 g) in the first period, and ribaxamase (150 mg) and ceftriaxone (1 g) in the presence of esomeprazole in the second period. In both periods, the concentrations of ceftriaxone were measured in plasma and chyme, and the concentrations of ribaxamase were measured in chyme. Both studies enrolled male and female subjects 18 and 80 years of age who had functioning ileostomies but were otherwise free from clinically significant illness or disease.

### Study details. (i) Common procedures.

In both studies, subjects who satisfied the screening criteria were admitted to the clinical research unit (CRU) on the day before period 1 of each study and underwent confirmatory eligibility assessments and an overnight stay. Samples for safety laboratory tests were collected within 48 h before dosing. In each period (1 and 2) of each study, subjects underwent an overnight fast of at least 8 h prior to the day of dosing and remained confined to the CRU until completion of the study procedures. On each day of dosing (periods 1 and 2) in each study, subjects received a small, nonfatty meal approximately 1 h before administration of i.v. ceftriaxone and again 5 h after the start of ceftriaxone infusion (Fig. 1). Subjects also received full meals at approximately 2 and 7.5 h after the start of the infusion. In order to help generate sufficient chyme output, subjects were required to drink water or apple juice at the start of the infusion and again at 0.5, 1, and 1.5 h after the start of the infusion; thereafter, subjects were encouraged to drink water or apple juice hourly after the start of the infusion. Serial blood and chyme samples were collected from approximately 30 min before through approximately 7 h for blood and 8.5 h for chyme (if present in the ileostomy bag) after the start of infusion for determination of ceftriaxone and ribaxamase concentrations.

In both studies, safety assessments consisted of clinical laboratory measurements, vital signs, electrocardiograms, physical examinations, and monitoring for adverse events (AEs). AEs were monitored from the time of informed consent signature until the end of study visit.

### (ii) Study 1 procedures.

For study 1 (Fig. 1A), eligible subjects were randomly assigned to one of two dose strengths of ribaxamase (75 or 150 mg/dose) at a 1:1 ratio. During period 1, all subjects only received a 30-min i.v. infusion of 1 g of ceftriaxone. Periods 1 and 2 were separated by a washout period of 3 to 7 days during which the subjects were released from the CRU. In the second period, all subjects received a 30-min i.v. infusion of 1 g of ceftriaxone and two oral doses (given 6 h apart) of one of the two dosage strengths of ribaxamase, according to the randomization schedule. The ribaxamase doses were administered 30 min before and 5.5 h after the start of the ceftriaxone infusion.

### (ii) Study 2 procedures.

In the second study during period 1 (Fig. 1B), all subjects received two oral doses of 150 mg of ribaxamase (with the same timing as in period 2 of study 1) and 1 g of ceftriaxone infused i.v. over 30 min. Periods 1 and 2 were separated by a run-in phase, during which the subjects self-administered esomeprazole (40 mg) once a day in the morning for 5 to 7 consecutive days. In period 2, subjects continued their esomeprazole dosing including a dose at ∼1.5 h before the start of the ceftriaxone infusion, and then ribaxamase and ceftriaxone were administered in the same manner as for period 1.

### Pharmacokinetic sample collection and assessment. (i) Sample collection.

Blood samples were collected during both periods of both studies 30 min before and serially up to 7 h after starting the i.v. ceftriaxone infusion for measurement of ceftriaxone concentrations in plasma. In study 1, period 2 only, blood samples were also collected for measurement of possible ribaxamase concentrations in plasma. All blood samples were immediately chilled and centrifuged using a refrigerated centrifuge to obtain plasma. For ceftriaxone analysis only, 1-ml aliquots of plasma were placed into tubes containing 10 μl of tazobactam solution (Sigma-Aldrich) at 10 mg/ml in sterile water to neutralize any possible, low-level, residual β-lactamase activity in the plasma sample (36). Plasma samples were stored frozen at −70°C until analyzed.

Samples of chyme (at least 2 ml, if present in the ileostomy bag at the sampling time) were collected during both periods of both studies at approximately 30 min before and serially for 8.5 h after starting the ceftriaxone infusion for assessment of ceftriaxone and ribaxamase concentrations. The total volume of chyme in the ileostomy bag was collected in 50-ml conical tubes, flash frozen in dry ice-ethanol, and stored frozen at −70°C until analyzed. A new ileostomy bag was then put in place.

### (ii) Chyme sample processing.

At the central lab, chyme samples were thawed in an ice bath and then vigorously vortexed and centrifuged in a refrigerated centrifuge to remove solids. The supernatant was aliquoted into prechilled tubes and an equal volume of 8 M guanidine HCl in 100 mM ammonium bicarbonate was added to the aliquots to be analyzed for ceftriaxone in order to neutralize any residual β-lactamase activity in the chyme supernatant. Chyme supernatants for analysis of ribaxamase concentrations were not treated with guanidine.

### (iii) Sample analysis.

Ceftriaxone concentrations were determined in chyme supernatant and plasma samples of both studies using a fully validated (plasma) or qualified (chyme) liquid chromatography turbo ion spray-tandem mass spectrometry method (LC-MS/MS). Plasma samples containing ceftriaxone were protein precipitated using acetonitrile and then separated on a Phenomenex Synergi Polar-RP, 4 μM, 2×50 mm (P/N 00B-4336-B0) column using 1% formic acid in water and 1% formic acid in methanol as the mobile phases. Standards and quality control samples were made by spiking ceftriaxone into pooled human plasma. Ceftriaxone was detected using an AB Sciex API 4000, positive ionization. [^13^C, ^2^H_3_]ceftriaxone was used as the internal standard. The lower limit of quantitation (LLOQ) for ceftriaxone concentration in plasma was 100 ng/ml. Chyme supernatants were similarly analyzed for ceftriaxone, except that a Phenomenex Synergi 4 μM Polar RP 80-Å column was used for separation of the sample, and quality control samples were made in naive human chyme supernatant (obtained under a separate clinical protocol by Synthetic Biologics). Standards were made in a surrogate matrix (FaSSIF buffer; BioRelevant, London, United Kingdom). The LLOQ for ceftriaxone concentration in chyme was 1.0 μg/ml.

Ribaxamase concentrations in plasma and chyme supernatants were determined using immunodetection assays. For study 1 period 2 only, plasma samples were analyzed for their concentrations of ribaxamase using a validated electrochemiluminescence (ECL) assay procedure as previously described (36). The LLOQ of the method was 0.8 ng/ml. Esomeprazole was found to interfere with the analysis of ribaxamase in human plasma in this assay (data not shown), so plasma samples from study 2 were not collected or analyzed for ribaxamase concentrations. Ribaxamase concentrations in chyme supernatant were also determined using a similar qualified ECL assay. FaSSIF buffer was again used for preparing standards, and quality control samples were prepared in human intestinal chyme supernatant. The LLOQ for ribaxamase concentrations in chyme was 10.0 ng/ml.

### Pharmacokinetic analysis.

The model-independent PK parameters of *C*_max_ (maximum observed concentration, by inspection without interpolation), *t*_max_ (time postdose of maximum observed concentration), and AUC_t_ (area under the concentration versus time profile to the last time point with a concentration greater than the assay LLOQ, calculated using the trapezoidal rule) were computed and reported for ceftriaxone in plasma. Since no ribaxamase was detected in any plasma sample, no parameters were calculated for this analyte. For concentrations of ceftriaxone and ribaxamase in chyme, no parameters were computed; only concentration data were reported.

### Statistical analysis.

Two populations were considered in the statistical analysis of the two studies: the safety population, which consisted of all subjects who had received at least one dose of ceftriaxone, and the PK analysis population, which consisted of all enrolled subjects who had completed at least 7 h of plasma ceftriaxone sampling, who did not have any major protocol deviations, and who were evaluable for at least the *C*_max_ or area under the concentration-time profile from time zero to 7 h after the start of the ceftriaxone infusion (AUC_0–7_) of plasma ceftriaxone in both periods 1 and 2.

No formal statistical tests were performed for the safety analyses for the study; review of data sets and summary statistics was used for evaluations. Safety summaries included TEAEs, which were defined as any event that began on or after the date of the first dose of any study drug or worsened in severity or frequency after dosing was initiated. Events worsening in severity were considered new AEs. The denominator used for calculation of the percentages was the number of subjects in the safety population per treatment per period.

Missing parts of dates were imputed and laboratory test values inconsistent with expected data type were subject to AE coding. Data from subjects with a protocol violation were not treated differently for safety analysis. Other missing data for AEs and laboratory values were not imputed. Missing PK data were treated as missing, and no imputation of the missing PK data was made. Concentrations below the assay LLOQ were set to zero.

## Supplementary Material

Supplemental material
