# Supplementary material for: The Oral β-Lactamase SYN-004 (Ribaxamase) Degrades Ceftriaxone Excreted into the Intestine in Phase 2a Clinical Studies
Source: Antimicrob Agents Chemother. 2017 Feb 23;61(3):e02197-16. doi: 10.1128/AAC.02197-16 (PMC5328510; doi:10.1128/AAC.02197-16)
Supplement: Supplemental material [file supp_61_3_e02197-16__index.html]

The Oral β-Lactamase SYN-004 (Ribaxamase) Degrades Ceftriaxone Excreted into the Intestine in Phase 2a Clinical Studies — Supplemental material 

# The Oral β-Lactamase SYN-004 (Ribaxamase) Degrades Ceftriaxone Excreted into the Intestine in Phase 2a Clinical Studies

## Supplemental material

- Supplemental file 1 -

  Tables S1 and S2 and Fig. S1 to S3

  PDF, 182K
