# Supplementary material for: The Oral β-Lactamase SYN-004 (Ribaxamase) Degrades Ceftriaxone Excreted into the Intestine in Phase 2a Clinical Studies
Source: Antimicrob Agents Chemother. 2017 Feb 23;61(3):e02197-16. doi: 10.1128/AAC.02197-16 (PMC5328510; doi:10.1128/AAC.02197-16)
Supplement: Supplemental material [file AAC.02197-16_zac003175977s1.pdf]

Table S1: Demographics and Baseline Characteristics of Enrolled Subjects

		Study 1<br>N = 11	Study 2<br>N = 15
Age (years)	Mean (SD)	48.5 (13.8)	45.1 (16.4)
	Median	46	46
	Range (Min, Max)	23, 74	22, 74
Ethnicity, n (%)	Not Hispanic or Latino	11 (100)	15 (100)
Race, n (%)	Other	1 (9.1)	1 (6.7)
	White	10 (90.9)	14 (93.3)
Sex, n (%)	Male	8 (72.7)	11 (73.3)
	Female	3 (27.3)	4 (26.7)
Height (cm)	Mean (SD)	173.0 (8.4)	172.1 (11.5)
	Median	173.0	171.5
	Range (Min, Max)	161.0, 188.0	152.0, 190.5
Weight (kg)	Mean (SD)	86.7 (18.4)	82.0 (19.3)
	Median	89.7	81.9
	Range (Min, Max)	54.3, 108.9	45.7, 113.6
BMI (kg/m^2)	Mean (SD)	28.8 (4.8)	27.4 (4.8)
	Median	30.0	27.9
	Range (Min, Max)	20.7, 34.3	18.7, 36.0

Note: Percentage (%) was calculated with number of subjects enrolled as the denominator for each Study.

Height was measured at Screening. Weight and BMI were measured at Day -1 of Period 1.

Abbreviations: BMI = body mass index; cm = centimeter; kg = kilogram; m = meter; Max = maximum; Min = minimum; n or N = number; SD = standard deviation

Table S2: Incidence of All Treatment-Emergent Adverse Events Considered Related to Study Drug - Safety Population

	Study 1 (N = 11 [b])	Grade [a]	Study 2 (N = 15)	Grade [a]
Total # of TEAEs, n [b]	12	n/a [b]	8	n/a
Subjects with at least 1 TEAE, n (%)	7 (63.6)	n/a	5 (33.3)	n/a
Headache, n (%)	2 (18.2)	1[c]	2 (13.3)	1
Somnolence, n (%)	1 (9.1)	1	0	n/a
Discomfort, n (%)	1 (9.1)	3[c]	0	n/a
Feeling cold, n (%)	1 (9.1)	3[c]	0	n/a
Stoma site hemorrhage, n (%)	0	n/a	1 (6.7)	1
Pain in extremity, n (%)	1 (9.1)	1[c]	0	n/a
Abdominal pain/discomfort, n (%)	1 (9.1)	1	1 (6.7)	1
Nausea, n (%)	0	n/a	1 (6.7)	2
Infusion related reaction, n (%)	1 (9.1)	3[c]	0	n/a
Anxiety, n (%)	1 (9.1)	1[c]	0	n/a
Pollakiuria, n (%)	1 (9.1)	1	0	n/a
Postmenopausal hemorrhage, n (%)	0	n/a	1 (6.7)	1
Rhinorrhoea, n (%)	0	n/a	1 (6.7)	1
Erythema, n (%)	1 (9.1)	1[c]	0	n/a
Hot flush, n (%)	0	n/a	1 (6.7)	1

[a] Grade 1, Does not interfere with daily activities; Grade 2, Interferes with daily activities; no treatment required except acetaminophen; Grade 3, Prevents daily activities or requires treatment; Grade 4, Life threatening

[b] Abbreviations: N or n = number, n/a = not applicable, TEAE = treatment emergent adverse event

[c] Occurred during Period 1 when only ceftriaxone was administered

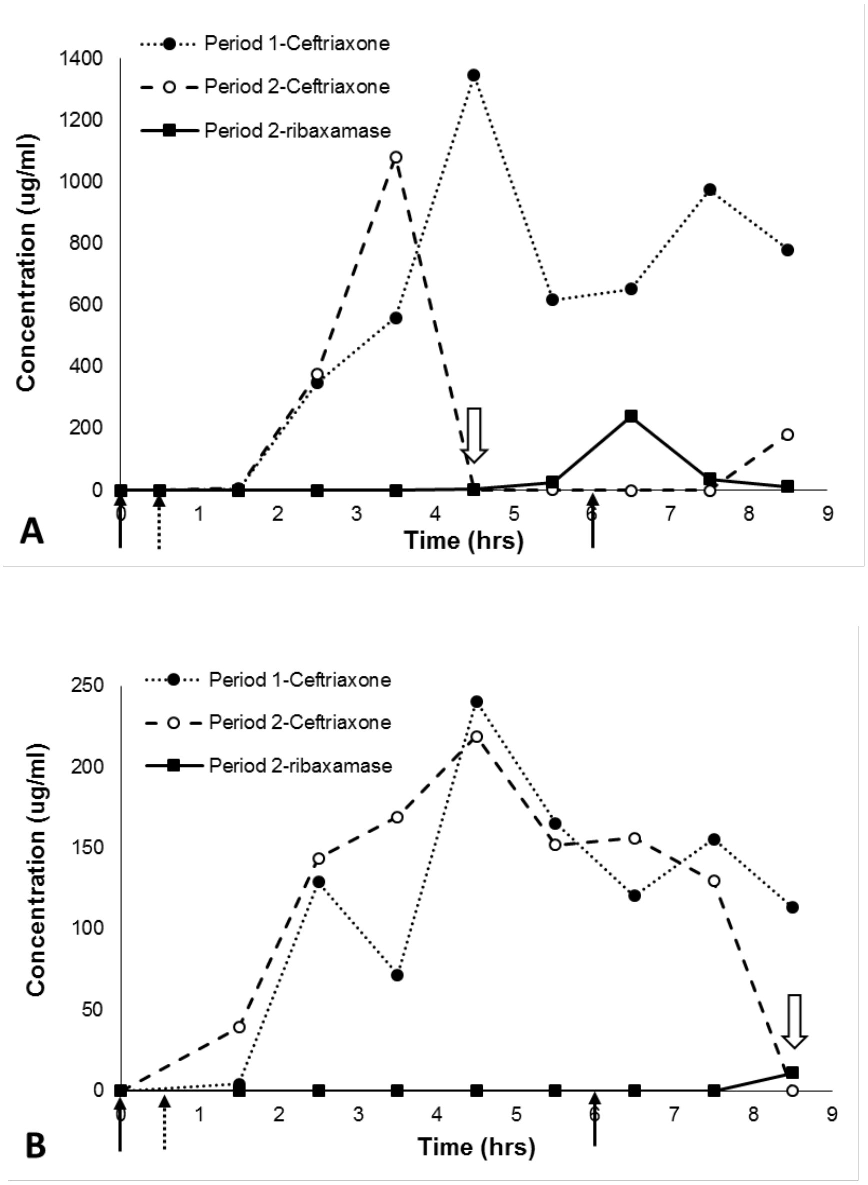

Figure S1 Intestinal Chyme Concentrations of Ceftriaxone and Ribaxamase for Two Subjects from Study 1. Data are presented for intestinal chyme concentrations of ceftriaxone and ribaxamase for two subjects who demonstrated delayed appearance of ribaxamase in their chyme during Period 2 of Study 1. The graphs display concentration curves for ceftriaxone (in Periods 1 and 2) and ribaxamase (Period 2 only) for Subjects 1006 (**A**) and 1011 (**B**). The 30 min ceftriaxone infusion began at the hatched arrow in Periods 1 and 2, while ribaxamase (150 mg and 75 mg respectively) was given orally at 0 and 6 h during Period 2 only (solid arrows). The open arrows are added for emphasis to indicate when ribaxamase was first detected in the chyme of these subjects during Period 2.

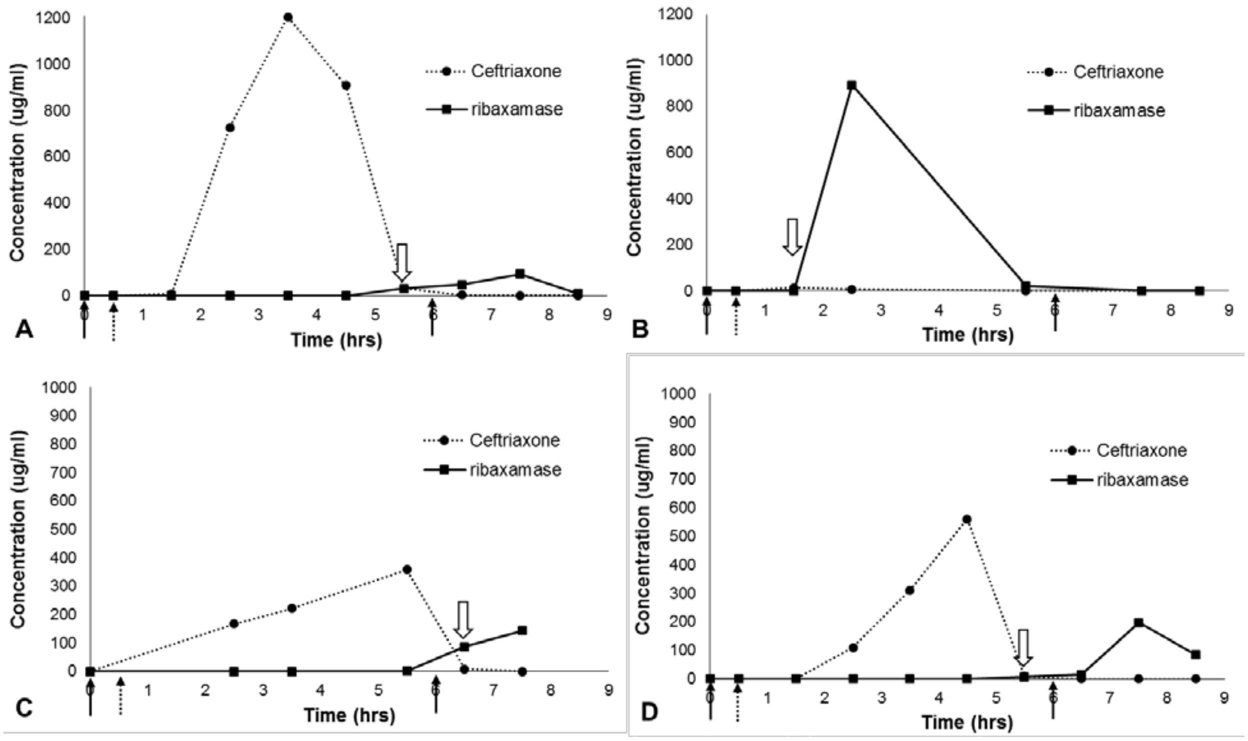

Figure S2 Intestinal Chyme Concentrations of Ceftriaxone and Ribaxamase for Two Subjects from Study 2. Data are presented for chyme concentrations of ceftriaxone and ribaxamase for two subjects who demonstrated delayed appearance of ribaxamase in their chyme during Study 2. The graphs display concentration curves for ceftriaxone and ribaxamase for Period 1 (**A** & **C**) and Period 2 (**B** & **D**) for Subjects 1002 (**A** & **B**) and 1008 (**C** & **D**). The 30 min ceftriaxone infusion began at the hatched arrows, while ribaxamase (150 mg) was given orally at 0 and 6 h (solid arrows). Period 2 is when esomeprazole was present. The open arrows are added for emphasis to indicate when ribaxamase was first detected in the chyme of these two subjects during these two periods. Missing data points indicate that no chyme was available for collection at that time point.

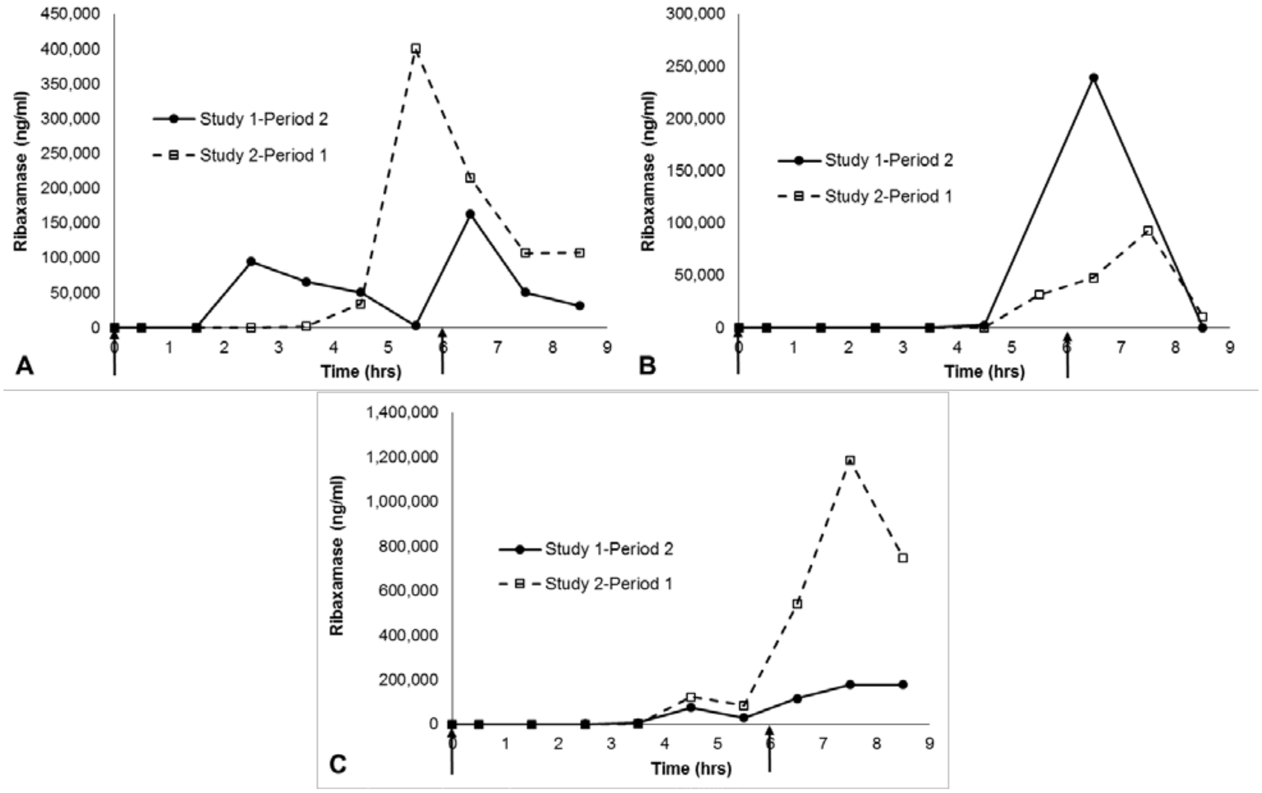

Figure S3 Comparison of Ribaxamase Concentrations in Intestinal Chyme for Three Subjects who Participated in Both Studies. Serial chyme samples (as available) were collected and analyzed for their ribaxamase concentrations over time in hours in Period 2 of Study 1 and Period 1 of Study 2 (as indicated on the figure). The data show individual concentration curves (assay lower limit of quantitation, 10 ng/ml) for three subjects who participated in both studies. Subject 1001/1001 (**A**) and Subject 1006/1002 (**B**) each received 150 mg of ribaxamase in both studies, while Subject 3009/2001 (**C**) received 75 mg ribaxamase in Study 1 and 150 mg ribaxamase in Study 2. (Subject numbers are shown as Study 1/Study 2.) The two doses of oral ribaxamase were administered at the solid arrows. Missing data indicate that no chyme was available for collection at that time point.
